# Structural and functional connectivity associations with anterior cingulate sulcal variability

**DOI:** 10.1007/s00429-024-02812-5

**Published:** 2024-06-20

**Authors:** Luke Harper, Olof Strandberg, Nicola Spotorno, Markus Nilsson, Olof Lindberg, Oskar Hansson, Alexander F. Santillo

**Affiliations:** 1 Clinical Memory Research Unit, Department of Clinical Sciences, Medical Sciences, Neuroscience, Lund University, Sölvegatan 19, 22100 Lund, Sweden; 2 Diagnostic Radiology, Faculty of Medicine, Department of Clinical Sciences, Lund, Sweden; 3 Division of Clinical Geriatrics, Karolinska Institute, Stockholm, Sweden; 4 Memory Clinic, Skåne University Hospital, Lund, Sweden

**Keywords:** Cingulate, Paracingulate, Sulcation, Resting-state, Functional Connectivity

## Abstract

Sulcation of the anterior cingulate may be defined by presence of a paracingulate sulcus, a tertiary sulcus developing during the third gestational trimester with implications on cognitive function and disease. In this cross-sectional study we examine task-free resting state functional connectivity and diffusion-weighted tract segmentation data from a cohort of healthy adults (< 60-year-old, n = 129), exploring the impact of ipsilateral paracingulate sulcal presence on structural and functional connectivity. Presence of a left paracingulate sulcus was associated with reduced fractional anisotropy in the left cingulum bundle and the left peri-genual and dorsal bundle segments, suggesting reduced structural organisational coherence in these tracts. This association was not observed in the offsite temporal cingulum bundle segment. Left paracingulate sulcal presence was associated with increased left peri-genual radial diffusivity and tract volume possibly suggesting increased U-fibre density in this region. Greater network dispersity was identified in individuals with an absent left paracingulate sulcus by presence of a significant, predominantly intraregional, frontal component of resting state functional connectivity which was not present in individuals with a present left paracingulate sulcus. Seed-based functional connectivity in pre-defined networks was not associated with paracingulate sulcal presence. These results identify a novel association between sulcation and structural connectivity in a healthy adult population with implications for conditions where this variation is of interest. Presence of a left paracingulate sulcus appears to alter local structural and functional connectivity, possibly as a result of the presence of a local network reliant on short association fibres.

**Supplementary Information:**

The online version contains supplementary material available at 10.1007/s00429-024-02812-5.

## Introduction

**Fig. 1:**
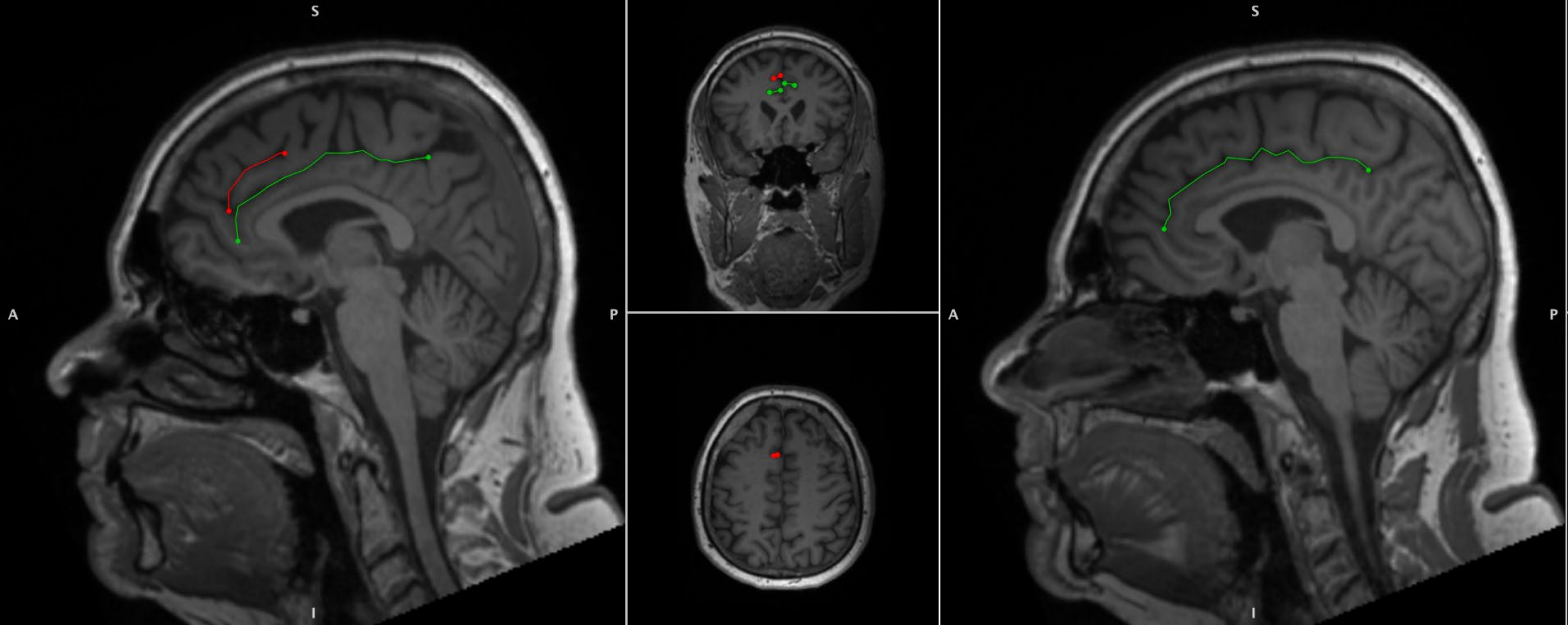
Cingulate and paracingulate sulci identification and measurement. 52-year-old male displaying a Leftward pattern of paracingulate asymmetry. Left panel, a sagittal slice of the left hemisphere with a traced “present” (length ≥ 20 mm), left paracingulate sulcus (red) and a traced left cingulate sulcus (green). Right panel, a sagittal slice of the right hemisphere, the paracingulate sulcus is absent here and only the cingulate sulcus is traced (green)

The Anterior Cingulate (AC) is a highly heterogenic medial frontal lobe gyrus with extensive interindividual variability and asymmetry. Variability may be classified according to the presence of a Paracingulate Sulcus (PCS), a tertiary sulcus, which when present develops during the third trimester of gestation and remains stable thereafter, unaffected by maturation or environmentally induced neuroplastic changes (Chi et al. 1977; Del Maschio et al. 2019). The PCS denotes the existence a respective Paracingulate Gyrus (PCG). In healthy individuals there is an established leftwards-dominance of PCS presence (presence of a left, but not right hemisphere PCS), as displayed in Fig. 1. (Paus et al. 1996; Yücel et al. 2001, 2002; Le Provost et al. 2003; Huster et al. 2007; Leonard et al. 2009; Wei et al. 2017; Amiez et al. 2018, Selahi et al. 2022). Whilst reported frequencies vary PCS are present in approximately 70–75% of left hemispheres and 50–60% of right hemispheres in the healthy population (Paus et al. 1996; Yücel et al. 2001; Fornito et al. 2004). The PCG is active during performance of a variety of cognitively demanding tasks drawing on higher-order executive function. (Fornito et al. 2004; Carter et al. 1998; Duncan and Owen 2000). A performance advantage across several verbal and non-verbal higher-order functions utilising effortful cognitive control and verbal and spatial working memory has been observed in individuals possessing a leftward asymmetry of PCS presence (Fornito et al. 2004; Whittle et al. 2009a, b). Similarly, individuals with asymmetric PCS patterns display greater inhibitory control and cognitive efficiency than those with symmetric patterns (Tissier et al. 2018; Borst et al. 2014a, b; Huster et al. 2009; Cachia et al. 2014; Fedeli et al. 2022). Bilateral PCS absence is considered cognitively disadvantageous and is associated with reduced reality monitoring and performance related introspection (Buda et al. 2011).

In disease PCS variability has been associated with schizophrenia and behavioural variant Frontotemporal Dementia (bvFTD), both of which have known pathological foci in the anterior cingulate. In bvFTD presence of a right PCS is associated with disease expression and survival (Harper et al. 2022, 2023), whilst a reduced frequency of leftward paracingulate sulcation dominance is observed in individuals with schizophrenia (Yücel et al. 2002; Le Provost et al. 2003; Yücel et al. 2003; Marquardt et al. 2005; Fujiwara et al. 2007). Furthermore, left hemisphere PCS presence is reportedly less frequent in patients with obsessive compulsive disorder (OCD; Shim et al. 2009) and altered PCG connectivity has been correlated with generalized epilepsy (Kay et al. 2014) and epilepsy drug resistance (Szaflarski et al. 2013; Wysiadecki et al. 2021).

There are several ways in which PCS presence may mediate function and thus its role in disease. Cortical folding, according to the tension-based morphogenesis theory is considered to be pathway specific, partially dependent on underlying tensions between short association fibres connecting neighbouring cortical regions which shorten to reduce wiring (Van Essen 1997, 2020). Reflecting this process, the PCG is connected structurally primarily by the contribution of U-fibres, connecting it with the AC proper forming a localised white matter network not present where the PCS is absent (Wysiadecki et al. 2021). These fibres may influence the observed underlying cytoarchitectural differences of the AC observed in the presence of a PCS (Vogt et al. 1995).

Deep of the superficial U-fibres lie longitudinal fibres which course within the PCG when present and are identified medial and slightly inferior to the cingulate sulcus, within the cingulate gyrus where the PCS is absent (Komaitis et al. 2019). This tract is regarded by some as subcomponent Ia of the superior longitudinal fasciculus (SLF), (Komaitis et al. 2019; Wysiadecki et al. 2021) and by others as a division of the cingulate bundle (Wu et al. 2016) or even U-fibres (Maldonado et al. 2012). To the best of our knowledge no neuroanatomical tracing or tractography studies have been performed with respect to PSC presence. From the perspective of intrinsically connected networks, the SLF-I is considered a major subcortical connection of the default mode network (DMN), a resting state network activated when the brain is resting (but alert) and attention is focused on internal tasks such as memory retrieval and self-reflection (Yagmurlu et al. 2016). Moreover, the AC contains the cingulum bundle and represents a key hub of the salience network (SN; Seeley et al. 2007). Operationally, the SN processes relevant stimuli by integrating sensory, emotional, and cognitive information, becoming active during tasks requiring attentional selection, task switching, and self-regulation of behavior (Farb et al. 2013; Fedeli et al. 2020; Seeley et al. 2007).

Presence of a PCS has been identified to alter the loci of task-based functional connectivity in numerous works (Jahn et al. 2016; Amiez et al. 2013; Loh et al. 2017). Resting-state functional connectivity with respect to the PCS has been examined in two studies (Fedeli et al 2020; Loh et al. 2017). In the first, Fedeli et al 2020 identified an association between PCS presence and functional connectivity in target voxels overlapping components of the SN and DMN but without the emergence of a convincing pattern of connectivity (Fedeli et al. 2020). In the second, Loh et al. 2018 studied anatomo-functional organization in left hemisphere cingulate motor ROIs in midcingulate cortex zones and observed similar organization in individuals with present and absent PCS (Loh et al. 2017). The impact of PCS presence on resting state functional connectivity remains incompletely explained and has not, to the best of our knowledge been studied alongside structural connectivity in the same cohort. In the present study we examine task-free resting state functional data and diffusion-weighted tract segmentation data in a cohort of young adults (< 60-year-old), exploring the impact of PCS presence on structural and functional connectivity.

With respect to the tension-based morphogenesis hypothesis of gyrification, (Van Essen 1997, 2020) structurally we hypothesise that presence of a PCS shall alter the SLF-I and/or cingulum bundle tracts both at a macroscopic (i.e., volume of the tracts) and microscopic (i.e., using diffusion tensor imaging metrices as proxies) level reflecting different local structural connectivity in individuals possessing a PCS. Functionally, we hypothesise that individuals with a present PCS shall display increased intraregional and decreased interregional connectivity relative to individuals with an absent PCS. Furthermore, we predict that individuals possessing a PCS shall display increased network connectivity in pre-defined local networks, the SN and DMN.

## Materials and methods

### Participants

In this retrospective analysis we studied data from healthy subjects from the Swedish BioFINDER-2 study (Skåne University Hospitals, Sweden [NCT03174938]), which was approved by the Regional Ethical Committee in Lund, Sweden, (EPN file number 2016/1053). Participants were enrolled between 2014 and 2021 following attainment of written consent in accordance with the Declaration of Helsinki. For further study details, see http://biofinder.se and (Palmqvist et al. 2020). Briefly, study participants were recruited using the following inclusion criteria: (i.) absence of cognitive symptoms, (ii.) Mini-Mental State Examination (MMSE) score of 26–30 at baseline, (iii.) not fulfilling criteria for mild cognitive impairment or dementia according to DSM-5 (American Psychiatric Association 2013), (iv.) absence of active psychological or psychiatric disease and (v.) fluency in Swedish. A total of 333 met these inclusion criteria and had available structural MRI data. Additional exclusion criteria applied in the present study were: (i.) age ≥ 60 years old, (ii.) an abnormal CSF amyloid-ß42/40 ratio, described in the Supplementary Material, (iii.) a high volume of white matter hyperintensities, (> 3 standard deviations from the cohort mean), described further in the Supplementary Material, and (iv.) poor MRI image quality obscuring identification of the PCS. After applying these procedures and following quality control measures, we included 129 individuals with available rsfMRI data in the function connectivity study and 125 individuals with available tract segmentation data in the structural connectivity study. Notably, 122 individuals participated in both studies. Demographic data is reported in Tables 1 and 2.

**Table 1:** Structural connectivity study population & paracingulate sulcal status

	Total	Left PCS present	Left PCS absent	Left PCS contrasts	Right PCS present	Right PCS absent	Right PCS contrasts
n	125	88	37		71	54	
Age, mean (SD), years	52.19 (5.12)	52.37 (5.06)	51.76 (5.33)	F = 0.36, *P* = 0.55	51.63 (4.95)	52.92 (5.30)	F = 1.94, *P* = 0.17
Sex				X^2^ = 3.15, *P* = 0.08			X^2^ = 1.95, *P* = 0.16
Female	71	45	26		36	36	
Male	54	43	11		35	19	
Handedness^a^				X^2^ = 1.09, *P* = 0.58			X^2^ = 1.67, *P* = 0.43
Right	113	80	33		64	49	
Left	8	5	3		4	4	
Ambidextrous	2	2	0		2	0	
Unknown	2	1	1		1	1	

Demographic data for the structural connectivity analyses

Standard deviation (SD). ^a^ Handedness data available for 123/125 individuals. Hemispheric Paracingulate Sulcal Status; present = PCS length ≥ 20 mm. ANOVA and Chi-Squared tests were performed to evaluate differences in continuous and nominal data, respectively

**Table 2:** Functional connectivity study population & paracingulate sulcal status

	Total	Left PCS present	Left PCS absent	Left PCS contrasts	Right PCS present	Right PCS absent	Right PCS contrasts
n	129	92	37		74	55	
Age, mean (SD), years	52.46 (4.96)	52.59 (4.95)	52.14 (5.05)	F = 0.22, *P* = 0.64	51.82 (4.79)	53.32 (5.11)	F = 2.95, *P* = 0.08
Sex				X^2^ = 3.61, *P* = 0.06			X^2^ = 1.00, *P* = 0.32
Female	72	46	26		38	34	
Male	57	46	11		36	21	
Handedness^a^				X^2^ = 0.83, *P* = 0.66			X^2^ = 2.11, *P* = 0.35
Right	116	83	33		67	49	
Left	9	6	3		4	5	
Ambidextrous	2	2	3		2	0	
Unknown	2	1	1		1	1	

Demographic data for the functional connectivity analyses

Standard deviation (SD). ^a^ Handedness data available for 127/129 individuals. Hemispheric Paracingulate Sulcal Status; present = PCS length ≥ 20 mm. ANOVA and Chi-Squared tests were performed to evaluate differences in continuous and nominal data, respectively

### Magnetic resonance image acquisition

MRI scans were performed on a MAGNETOM Prisma 3 T scanner (Siemens Healthineers, Erlangen, Germany), equipped with a 64-channel head coil. A T1w MPRAGE (magnetization-prepared rapid gradient-echo) sequence was acquired with the following acquisition parameters: repetition time: 1900 ms; echo time: 2.54 ms; echo spacing: 7.3 ms; voxel size: 1 × 1 × 1 mm^3^ and field of view: 256 × 256 × 176 mm^3^. GRAPPA (generalized autocalibrating partially parallel acquisitions^33^) was applied with acceleration factor of 2 and 24 reference lines. A single-shot echo-planar imaging sequence was used to acquire 104 diffusion-weighted imaging volumes (repetition time: 3500 ms; echo time: 73 ms; resolution: 2 × 2 × 2 mm^3^; field of view 220 × 220 × 124 mm^3^; b values range: 0, 100, 1000, and 2500 s/mm^2^ distributed over 2, 6, 32, and 64 directions; twofold parallel acceleration and partial Fourier factor = 7/8). A second diffusion MRI scan was also obtained with a reverse phase-encoding and 7 gradient directions (1 × b = 0 and 6 × b = 1000 s/mm^2^) for correction of susceptibility-induced distortions. A T2-weighted FLAIR scan (repetition time: 5000; echo time 393 ms, same resolution and FoV as for the T1-weighted image) was also acquired. Spontaneous blood oxygen level-dependent (BOLD) oscillations were acquired with a gradient-echo planar sequence (eyes closed, in-plane resolution = 3 × 3 mm^2^, slice thickness = 3.6 mm, repetition time = 1020 ms, echo time = 30 ms, flip-angle = 63°, 462 dynamic scans, 7.85 min).

### Paracingulate sulcus measurement and classification criteria

Individuals were grouped in accordance with hemispheric presence of a PCS. PCS presence was identified via manual classification of structural T1 MRI data according to an adapted version or Garrison’s established protocol for PCS classification (Garrison et al. 2015), which has been used and described previously (Harper et al. 2022, 2023) and is documented in full in the Supplementary Material. Potential PCS, meeting classification criteria were manually traced and measured. Briefly, the PCS is identified as the sulcus running predominantly horizontally, dorsal, and parallel to the Cingulate Sulcus (CS). The PCS is measured from its anterior limit, identified as the point at which the sulcus begins to move posteriorly and parallel to the CS from an imaginary line perpendicular to the bicommissural (AC-PC) line (Yücel et al. 2001). The PCS is measured from this point until its end, the point at which the sulcus is interrupted by a distinct predominantly vertical gyri, deemed non-PC in nature. The PCS may fall outside of the first quadrant but must originate within the first quadrant on a sagittal plane where × 0, y0 marks the point of the anterior commissure. As is standard amongst classification protocols hemispheres with a PCS ≥ 20 mm in length were categorised as possessing a “present” PCS whereas hemispheres failing to meet these criteria were deemed to possess an “absent” PCS (Ono et al. 1990, Yücel et al. 2002; Le Provost et al. 2003; Garrison et al. 2015; Del Maschio et al. 2019). Sulcation ratings were performed independently by two raters, LH and AS, who were blinded to individuals’ demographic data. Disagreement between raters was resolved by consensus.

### MRI data processing

MPRAGE images were imported into MANGO (Multi-image Analysis GUI, v 4.0, http://ric.uthscsa.edu/mango/mango.html, The University of Texas Health Science Centre) software and prepared, aligning the x axis in the sagittal plane with the AC–PC line. Further y and z axis rotational corrections were performed in order to ensure optimal orientation for analysis.

### Tract segmentation

Diffusivity metrics obtained from diffusion imaging data may be used to study white matter tract organisation (Alexander et al. 2011). Fractional anisotropy (FA) quantifies the degree of directionality of diffusion. Increased FA indicates highly directional diffusion, typically associated with increased microstructural integrity and tract organisation. Radial diffusivity (RD) meanwhile measures average diffusion perpendicular to the principal diffusion direction. Increased RD values may therefore indicate an increase in fibres running perpendicularly to a tract or de- or dysmyelination. Axial diffusivity (AD) measures the average diffusion along the primary axis of a tract and is sensitive to axonal integrity, diameter, and density. Finally, mean diffusivity (MD) is a measure of the average rate of diffusion within a tract. Increased MD indicates less restricted diffusion associated with reduced tract integrity. All metrics are sensitive to crossing of white matter tracts.

**Fig. 2:**
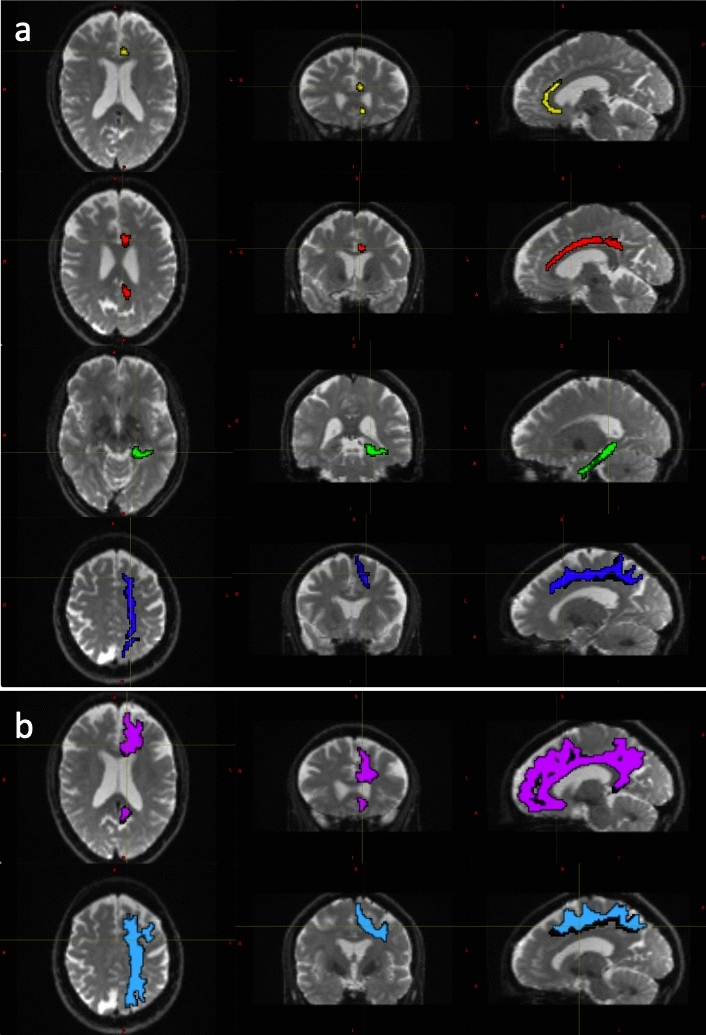
Tract Segmentations of the cingulate bundle and superior longitudinal fasciculus. Diffusion weighted image data for a 53-year-old female participant with left hemisphere tract segmentation mask overlays. **a** Displays the cingulum, divided into the peri-genual (CBG, yellow), dorsal (CBD, red) and temporal (CBT, green) tracts and the superior longitudinal fasciculus I (SLF-I, dark blue), extracted according to the Xtract definition. **b** Displays the cingulum bundle (CG, pink) and the superior longitudinal fascicules (SLF-I, light blue) extracted according to the Tract Seg definition

Diffusion weighted data were processed using a combination of open-source algorithms. In brief the acquired images were denoised and the Gibbs ringing artifacts were removed using *MRtrix3* (Tournier et al. 2019) routines. Correction for susceptibility induced distortions, using images acquire with opposite phase polarities, motion and Eddy currents was performed employing *FSL Top-up* (Andersson et al. 2003) and *Eddy* (Andersson and Sotiropoulos 2016; ﻿FMRIB Software Library, version 6.0.4; Oxford, United Kingdom). Parametric maps of MD, FA, AD and RD were computed using DIPY (Henriques et al. 2021) routines (https://dipy.org/). Following pre-processing of diffusion MRI scans, white matter tracts were segmented using, TractSeg (Wasserthal et al. 2018). Both the 72 tracts definition included in TractSeg and the 42 tracts definition derived from Xtract (Warrington et al. 2020) were used in order to improve internal validity. Furthermore, the Xtract method divides the cingulate bundle into three distinct tracts offering a more focused analysis of white matter contiguous with the PCS. Diffusivity metrics and tract volumes were analysed in accordance with ipsilateral hemispheric PCS presence in the superior longitudinal fasciculus I (SLF-I) [both segmentation methods], the cingulum (CG) [TractSeg] and the dorsal (CBD), pre-genual (CBG), and temporal (CBT) cingulum [Xtract]. Further method description and quality control measures are documented in the Supplementary Material. Tract segmentations examples are displayed in Fig. 2.

### Resting state functional MRI pre-processing

Resting state functional MRI data pre-processing was performed using a pipeline composed of FSL (Jenkinson et al. 2012), AFNI(Cox 1996) and ANTS(Avants et al. 2014). Anatomical processing involved skull stripping, segmentation of CSF, white and grey matter, and normalization to MNI152 space (Grabner et al. 2006). Following bulk motion and slice timing correction, nuisance regression compensated white matter/CSF signal, physiological noise (Behzadi et al. 2007), motion parameters (Johnstone et al. 2006), and scanner drift. Finally, the functional data were band-pass filtered (0.01–0.1 Hz) and transformed to MNI space. Frames causing outliers in total frame-to-frame signal variation (75 percentile + 1.5 interquartile range) were censored (Power et al. 2012). Subjects with a mean/maximum framewise displacement exceeding 0.3/3.0 mm were excluded. The processed functional MRI data were resampled to 6 × 6 × 6 mm^3^ and masked with grey matter derived from a cortical resting-state network atlas (Thomas Yeo et al. 2011), Harvard–Oxford subcortical atlas (Desikan et al. 2006). The variance stabilized Fisher z-transformed Pearson correlation between the resulting grey matter BOLD voxel time series yielded our functional connectivity measure.

### Statistical analysis

#### Tract segmentation analysis

Diffusivity metrics and tract volume analyses were performed in R software (R Version 4.2.1 CoreTeam 2016, https://www.r-project.org/) using general linear models, including age, sex, and handedness as covariates in all models. In addition, individual’s total intracranial volume was included as a covariate in all models analysing tract volume. As these analyses were explorative correction for multiple comparisons was not performed.

#### Seed-based functional connectivity analysis

The Salience/Ventral Attention (SN), Default mode (DMN) and Visual networks (VN) were defined geographically according to network parcel locations defined by the Schaefer 200 parcel 7 network atlas (Schaefer et al. 2017), further descriptions are provided in the Supplementary Material.

Functional connectivity (FC) analysis was performed using Pearson correlation coefficients between the mean time series of the 200 seeds corresponding to the 200 parcels of the Schaefer 200 parcel 7 network atlas. FC’s were converted into z-scores to improve normality using Fisher r-to-z transformation. Individuals z-scores were then averaged across ROIs relating to the predefined networks of interest. Finally, GLMs were fitted according to group averaged z-scores determined by ipsilateral PCS presence, controlling for the effects of age, sex, and handedness. Significance was identified at *P* = 0.05.

### Voxel-based functional connectivity analysis

A medial frontal lobe region of interest (ROI) was created for each hemisphere using the Schaefer 200 parcel 7 network atlas (Schaefer et al. 2018). Selected parcels were those overlapping the predicted location of the PCS in MNI-152 space (Grabner, Janke et al. 2006). ROIs are detailed in Supplementary Fig. 1.

Voxel wise whole brain connectivity in 6 × 6 × 6 mm space was evaluated using a two-step procedure. First the mean connectivity of all voxels was calculated using Persons r correlation.

**Fig. 3:**
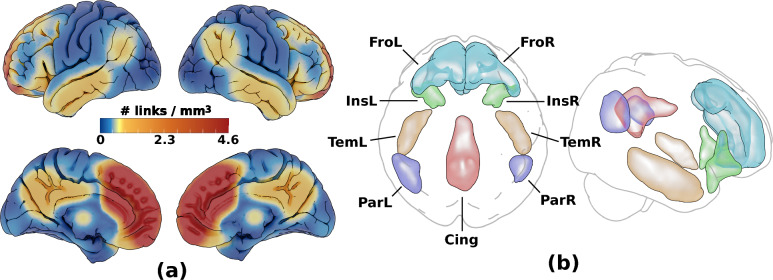
Voxel-based connectivity results. Cortical ROI set with high connectivity to the medial frontal lobe ROI, (visualised in Supplementary Fig. 2). **a** shows the link density from the medial frontal lobe ROI used to manually delineated highly connected regions in **b**: frontal (Fro: overlaps cing ant/mid, front sup med, front sup/mid, front mid/med/inf/sup orb), insular (Ins: overlaps insula, temp pole mid/sup, front inf orb/tri), temporal (Tem: overlaps temp inf/mid, temp pole mid), parietal (Par: overlaps par inf, temp mid/sup, angular, supramarginal) and posterior cingulate (Cing: overlaps cing mid/post, precuneus). To these, two subcortical ROIs defined in the Harvard–Oxford subcortical atlas were added (not shown): basal ganglia (BG: thalamus, Caudate, Pallidum, Putamen) and hippocampus/amygdala (HiAm)

The functional connectome was then restricted with a network mask corresponding to high connectivity with the medial frontal lobe ROIs by thresholding the all-subject-mean connectivity of all subjects at a correlation corresponding to *P* = 0.0001 (given the number of frames in the rs-fMRI time series). Cortical ROIs corresponding to this network mask, which included the bilateral anteromedial frontal cortices as well as portions of the insular, lateral temporal, parietal, and posterior cingulate cortices were then drawn on the resulting voxel-wise link density maps, see Fig. 3**.** These regions are part of the DMN and SN resting state networks, which both overlap with the source region. As scattered connectivity was obtained with subcortical regions of the basal ganglia and hippocampus/amygdala, these structures were added to the ROI set using the anatomical definitions according to the Harvard–Oxford subcortical atlas (Desikan et al. 2006) and not by manual delineation. Note that the tracing of these regions only affected the visualization and labelling in the resulting connectograms and that the network mask used in the calculation was applied to the links and not the voxels.

**Fig. 4:**
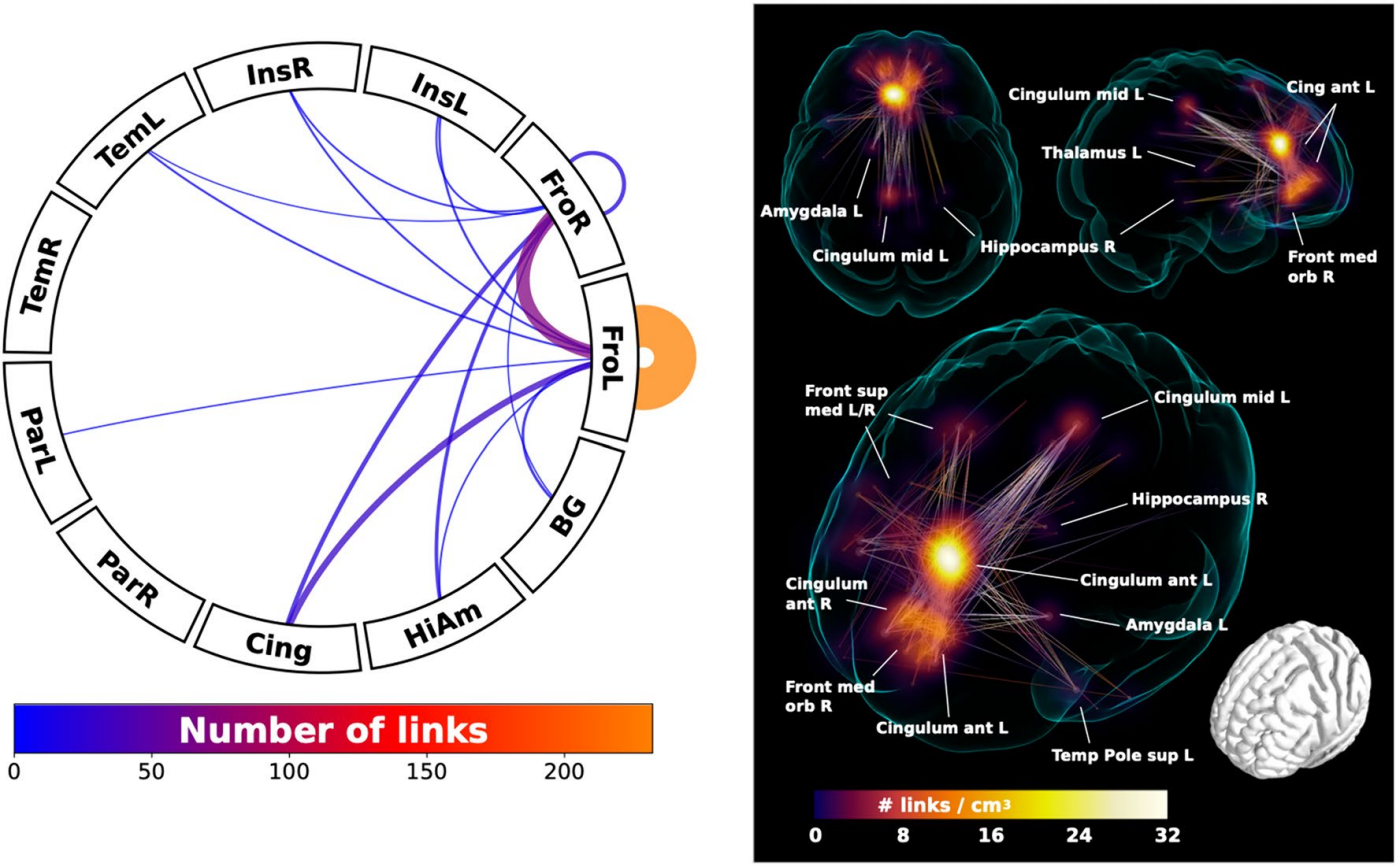
Voxel-based connectivity results. Network component with significantly greater functional connectivity in individuals with an absent left paracingulate sulcus relative to individuals with a present left paracingulate sulcus (*P* = 0.01 controlled for family-wise error rate). The left panel shows the connectogram in the form of the number of links connecting the left paracingulate ROI to highly connected regions (see Fig. 3). Number of links are represented by line thickness and colour, as denoted in the colour bar below. The right panel illustrates regions with the greatest link density (shown in voxel-wise maximal intensity projection) converging on the left anterior cingulate gyrus (Cingulum ant L), extending inferiorly towards the right frontal medial orbitofrontal (Front med orb R) and the right anterior cingulate gyrus (Cingulum ant R). More extended connections were also found to frontal superior medial gyrus L/R (Front sup med L/R) and the posterior cingulate gyrus (Cingulum mid L), as well as scattered connections to subcortical structures: left amygdala (Amygdala L), right posterior hippocampus (Hippocampus R), left thalamus (Thalamus L) and left superior temporal pole (Temporal Pole sup L). Lines are included to illustrate links between connected regions

In the second step, the whole brain functional connectome was limited to the identified regions connecting strongly to the ROIs and entered into a network based statistic component calculation (Zalesky et al. 2010) comprising a connected set of links on which connectivity differed in accordance with ipsilateral hemispheric PCS presence, based on a binarized connectivity graph at a threshold of *P* < 0.001 (given group sizes), controlled for the effects of age, sex and handedness. Results of significant network components with altered connectivity and summarizing connectograms are displayed in Fig. 4.

### Data availability

Anonymized data will be shared by request from a qualified academic investigator for the sole purpose of replicating procedures and results presented in the article if data transfer is in agreement with relevant legislation on the general data protection regulation and decisions and by the relevant Ethical Review Boards, which should be regulated in a material transfer agreement.

## Results

### Tract segmentation

Following quality control procedures segmentations were available for 125 subjects, (mean age 52.19, SD 5.12), see Table 1. The frequency of present to absent PCS was greater in the left (88/125) than right (71/125) hemisphere as expected.

Tract segmentation results are displayed in Table 3**.** Individuals with a present left PCS displayed reduced structural organisation of the CG (TractSeg), evidenced by a lower FA (β = − 0.02, CI − 0.01to −0.0008 µm^2^/ms, *P* = 0.02) than that observed in individuals with an absent left PCS. Using the Xtract method the cingulum is sub-divided into three divisions. Structural disorganisation was greatest in the CBG where presence of a left PCS was associated with decreased FA in the ipsilateral CBG (β = − 0.009, CI − 0.04to −0.008 µm^2^/ms, *P* = 0.002). Furthermore increased RD (β = 2.22 × 10^–5^, CI 7.58e−06—3.69e−05 µm^2^/ms, *P* = 0.003) and tract volume (β = 0.10, CI 0.02—0.18 µm^2^/ms, *P* = 0.012) was identified in the CBG in individuals with a present left PCS, indicating increased diffusion perpendicular to the principal diffusion direction and increased U-fibre presence.

**Table 3:** Structural connectivity results

Tract	Segmentation method	Tract volume	Fractional anisotropy	Mean diffusivity	Radial diffusivity	Axial diffusivity
Cingulum bundle, left	TractSeg	β = 0.50, *P* = 0.53	β = − 6.3e−03, *P* = 0.02*	β = 6.0e−06, *P* = 0.17	β = 8.0 e−06, *P* = 0.09	β = 1.9 e−06, *P* = 0.65
Cingulum bundle, right	TractSeg	β = 0.59, *P* = 0.30	β = − 4.2 e−04, *P* = 0.87	β = − 4.7 e−07, *P* = 0.91	β = − 5.7 e−07, *P* = 0.90	β = − 2.6 e−07, *P* = 0.95
Peri-genual cingulum, left	Xtract	β = 0.09, *P* = 0.01*	β = − 0.02, *P* = 0.002*	β = 1.1 e−05, *P* = 0.10	β = 2.2 e−05, *P* = 0.003*	β = − 1.1 e−05, *P* = 0.39
Peri-genual cingulum, right	Xtract	β = − 0.05, *P* = 0.28	β = 2.2 e−03, *P* = 0.72	β = − 6.4 e−06, *P* = 0.33	β = − 6.4 e−06, *P* = 0.33	β = − 3.4 e−07, *P* = 0.97
Cingulum bundle dorsal, left	Xtract	β = 0.29, *P* = 0.07	β = − 9.3 e−03, *P* = 0.03*	β = 5.7 e−06, *P* = 0.24	β = 6.7 e−06, *P* = 0.09	β = − 2.2 e−06, *P* = 0.72
Cingulum bundle dorsal, right	Xtract	β = 0.03, *P* = 0.82	β = − 4.5 e−03, *P* = 0.28	β = − 3.6 e−07, *P* = 0.94	β = 1.9 e−06, *P* = 0.73	β = − 4.9 e−06, *P* = 0.43
Cingulum bundle temporal, left	Xtract	β = 0.11, *P* = 0.33	β = − 2.9 e−03, *P* = 0.48	β = 1.6 e−05, *P* = 0.04*	β = 1.6 e−05, *P* = 0.04*	β = 1.6 e−05, *P* = 0.10
Cingulum bundle temporal, right	Xtract	β = 0.02, *P* = 0.83	β = − 3.5 e−03, *P* = 0.47	β = 3.0 e−07, *P* = 0.97	β = 2.2 e−06, *P* = 0.77	β = 1.6 e−05, *P* = 0.10
Superior longitudinal fasciculus I, left	TractSeg	β = − 0.81, *P* = 0.21	β = 5.5 e−06, *P* = 0.25	β = 7.1 e−06, *P* = 0.18	β = 7.1 e−06, *P* = 0.18	β = 2.3 e−06, *P* = 0.67
Superior longitudinal fasciculus I, right	TractSeg	β = 0.29, *P* = 0.71	β = 1.6 e−04, *P* = 0.96	β = − 3.7 e−06, *P* = 0.41	β = − 3.4 e−06, *P* = 0.49	β = − 3.5 e−06, *P* = 0.71
Superior longitudinal fasciculus I, left	Xtract	β = 0.12, *P* = 0.61	β = − 5.9 e−03, *P* = 0.23	β = 4.7 e−06, *P* = 0.44	β = 6.6 e−06, *P* = 0.35	β = 8.6 e−06, *P* = 0.90
Superior longitudinal fasciculus I, right	Xtract	β = 0.22, *P* = 0.34	β = − 2.1 e−03, *P* = 0.63	β = − 4.8 e−06, *P* = 0.39	β = − 2.2 e−06, *P* = 0.73	β = − 1.0 e−05, *P* = 0.18

General Linear Models for differences in tract structural connectivity matrices according to ipsilateral paracingulate sulcal presence. All models are corrected for age, sex, and handedness. Model of tract volume are additionally corrected for total intracranial volume. * Denotes significance at *P* < 0.05

Reduced tract organisation, indicated by lower FA was also identified in the left CBD in the presence of an ipsilateral PCS (β = − 0.009, CI – 0.02to − 0.0009 µm^2^/ms, *P* = 0.03). RD and tract volumes were however similar in this tract. In the offsite left CBT, left PCS presence was not associated with altered FA or tract volume, as expected. There were however small but significant increases in MD (β = 1.6e−05, P = 0.04) and RD (β = 1.6e−05, P = 0.04) in the CBT in individuals with a present PCS relative to individuals with an absent PCS, depicted in Supplementary Fig. 2.

**Fig. 5:**
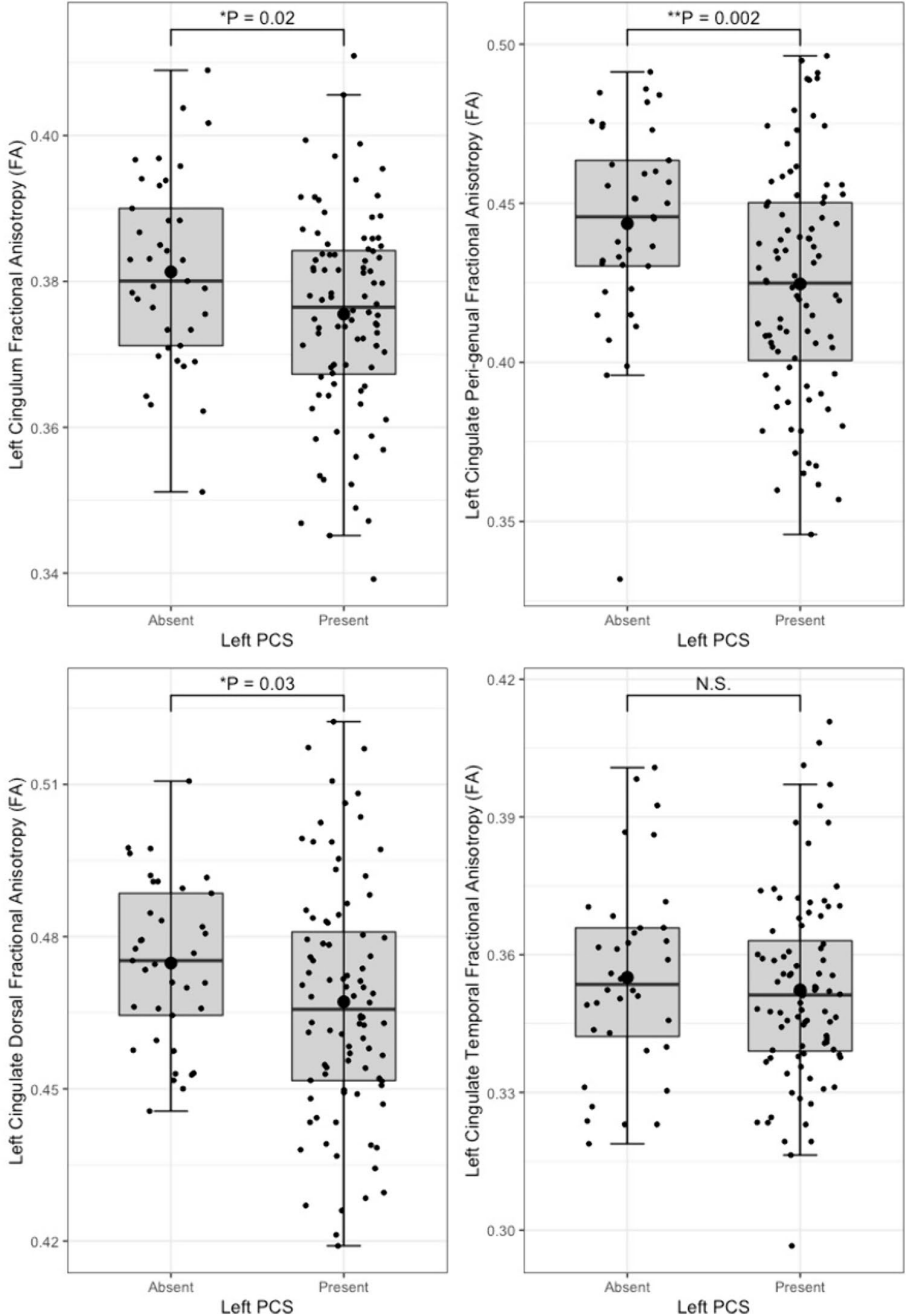
Cingulum bundle fractional anisotropy by left paracingulate sulcal presence. Box plots displaying tract fractional anisotropy by left Paracingulate sulcal presence in the cingulum [TractSeg] (top left), peri-genual cingulum [Xtract] (top right), dorsal cingulum [Xtract] (bottom left) and temporal cingulum [Xtract] (bottom right). Black dots represent individuals, n = 125. Thick horizontal black lines represent group median values. Larger black dots represent group mean values. Boxes extend from the 25th to the 75.^th^ percentile, horizontal black lines within the boxes denote median values. *P*-values (P) of general linear models corrected for age, sex and handedness are displayed over the box plots. N.S. denotes no significant difference between groups. * Significance at *P* =  < 0.05. ** P =  < 0.01

**Fig. 6:**
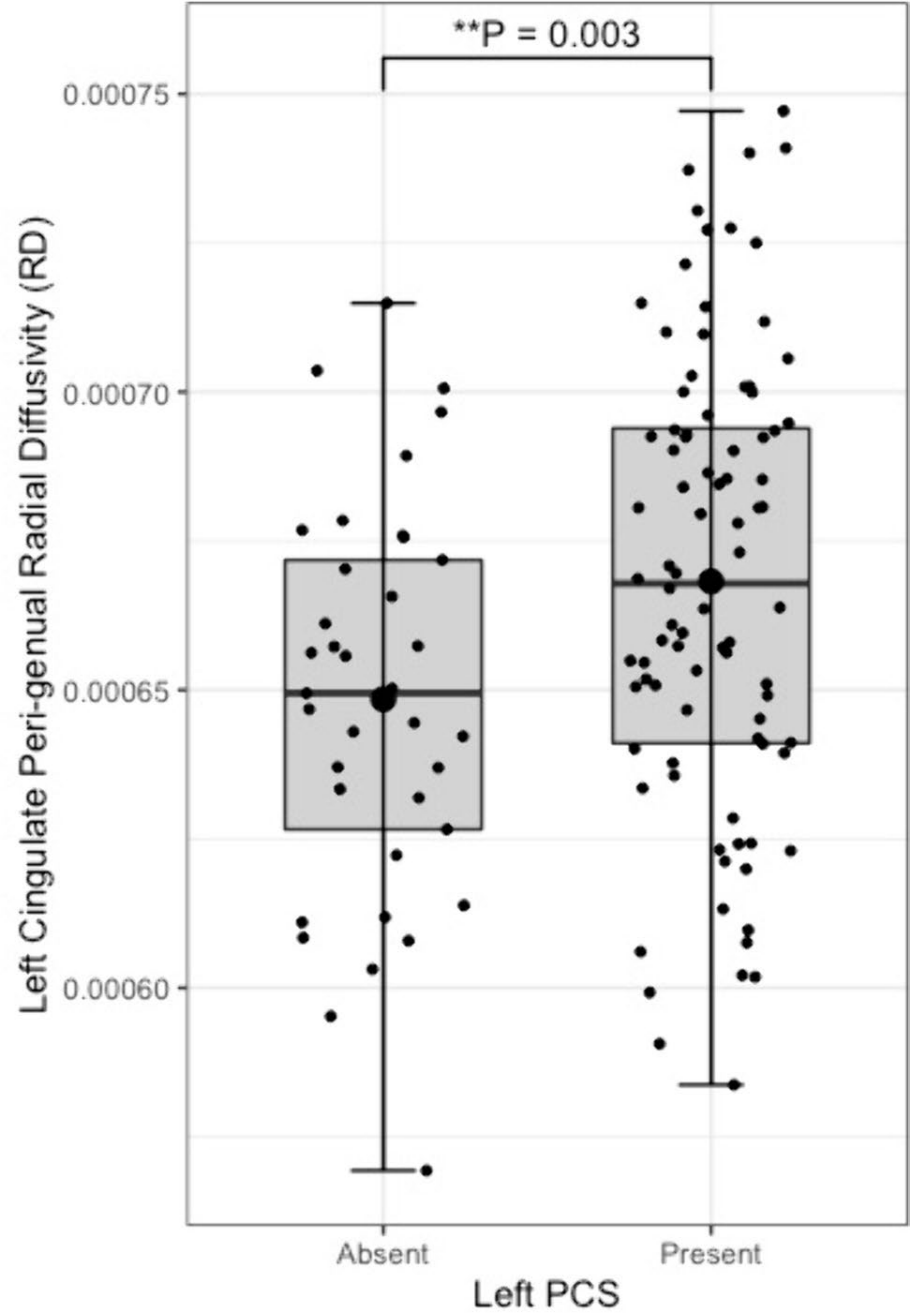
Peri-genual cingulum radial diffusivity by left paracingulate sulcal presence. Box plot displaying left peri-genual cingulum [Xtract] radial diffusivity by left Paracingulate sulcal presence. Black dots represent individuals, n = 125. Thick horizontal black lines represent group median values. Larger black dots represent group mean values. Boxes extend from the 25th to the 75.^th^ percentile, horizontal black lines within the boxes denote median values. P-values (P) of general linear models corrected for age, sex and handedness are displayed over the box plots. * Significance at P =  < 0.05. ** P =  < 0.01

**Fig. 7:**
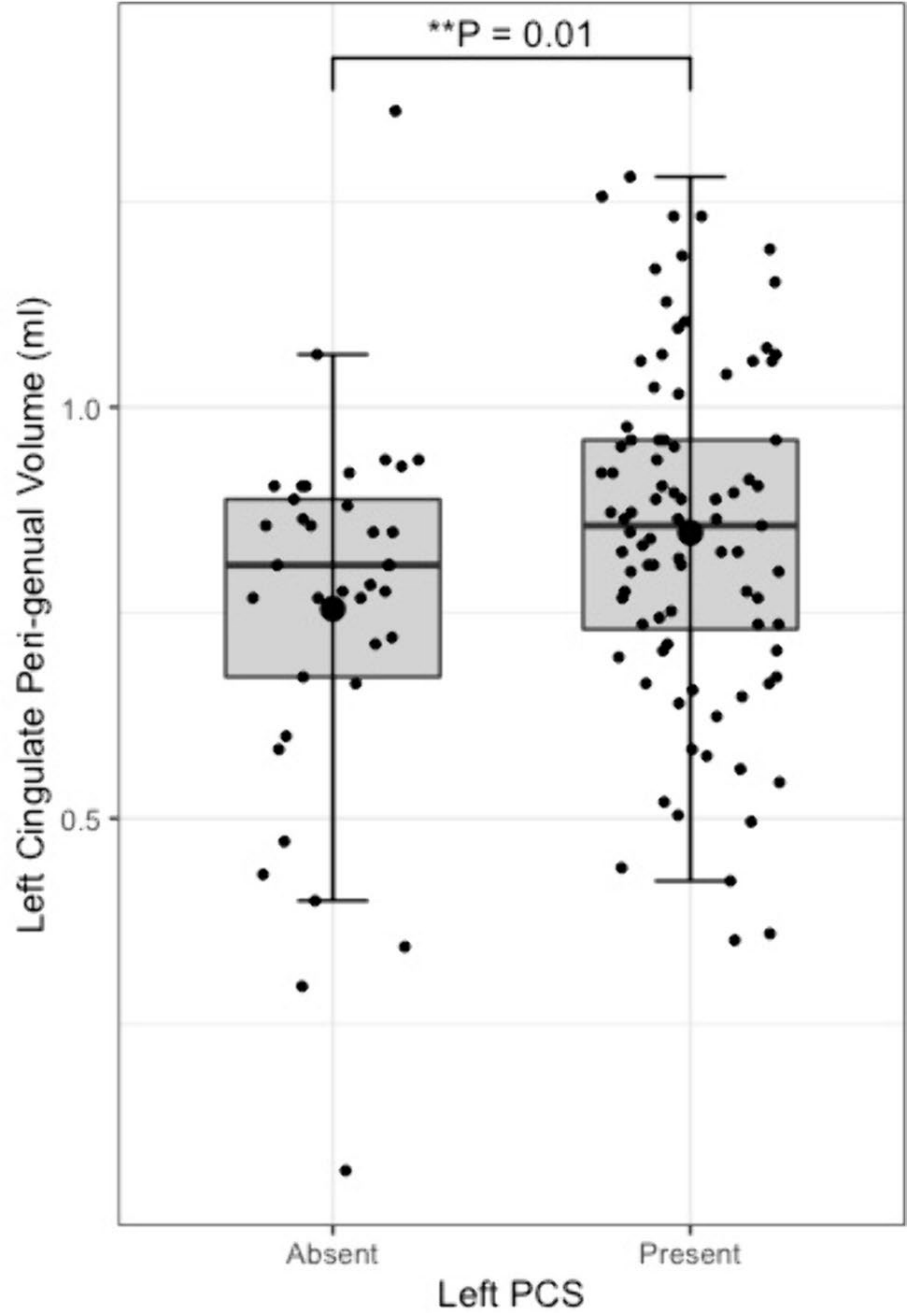
Peri-genual cingulum bundle tract volume by left paracingulate sulcal presence. Box plot displaying left peri-genual cingulum [Xtract] tract volume by left Paracingulate sulcal presence. Black dots represent individuals, n = 125. Thick horizontal black lines represent group median values. Larger black dots represent group mean values. Boxes extend from the 25th to the 75.^th^ percentile, horizontal black lines within the boxes denote median values. P-values (P) of general linear models corrected for age, sex and handedness are displayed over the box plots. * Significance at P =  < 0.05. ** P =  < 0.01

In the right hemisphere similar structural connectivity metrics and volumes were observed in the right CG, CBG, CBD and CBT independent of ipsilateral PCS presence. Neither left nor right PCS presence was associated with differences in metrics of ipsilateral structural connectivity or volume in the SLF-I. Key results may be visualised in Figs. 5, 6, 7.

### Functional connectivity

Resting state fMRI data was available for 129 individuals, (mean age 52.46, SD 4.96), see Table 2. Two individuals were excluded from analysis as they did not have available handedness data. Functional connectivity analyses were therefore performed on a population of 127 individuals.

### Seed-based functional connectivity

Group wise intra-network resting state functional connectivity (rsFC) in ipsilateral hemispheric and whole brain analyses of all predefined networks (SN, DMN and VN) were similar when comparing individuals with a present and absent left and right PCS. Results from GLMs are displayed in Supplementary Table 1.

### Voxel-based functional connectivity

A significant component representing a dispersed functional network was identified in individuals with an absent left PCS relative to individuals with a present left PCS at *P* = 0.01, controlling for family wise error rate. Results are displayed in Fig. 4 and Supplementary Fig. 3. The greatest link density was found converging on the left anterior cingulate gyrus, extending inferiorly towards the frontal medial orbitofrontal gyrus and the right anterior cingulate gyrus. More extended connections were also found to the frontal superior medial gyrus, the left and right posterior cingulate gyrus, as well as scattered connections to subcortical structures including the left amygdala, the right posterior hippocampus, and left thalamus.

## Discussion

Results from the tract segmentation analyses indicate that absence of a left hemisphere PCS is associated with higher ipsilateral cingulate bundle FA. More specifically group diffusivity differences localise to the anterior portion of the cingulum; the peri-genual and dorsal cingulum bundles. Furthermore, higher radial diffusivity and increased tract volume was observed in the left peri-genual cingulum bundle in individuals with a left PCS relative to those without. Expectedly, no significant group FA or volume differences were observed in the offsite temporal division of the cingulate bundle. In this context the marginally significant increases in MD and RD in the CBT of individuals possessing a left PCS are difficult to interpretate and may be explained by false discovery. Ipsilateral group tract volumes and diffusivity metrics were similar in the SLF-I in both hemispheres. These results suggest that where a left PCS is present the ipsilateral cingulum bundle, specifically its anterior portions (peri-genual > dorsal) may display increased orientational dispersion. To the best of our knowledge these findings are novel and an association between gyrification and structural connectivity in healthy individuals has not previously been identified in the literature.

**Fig. 8:**
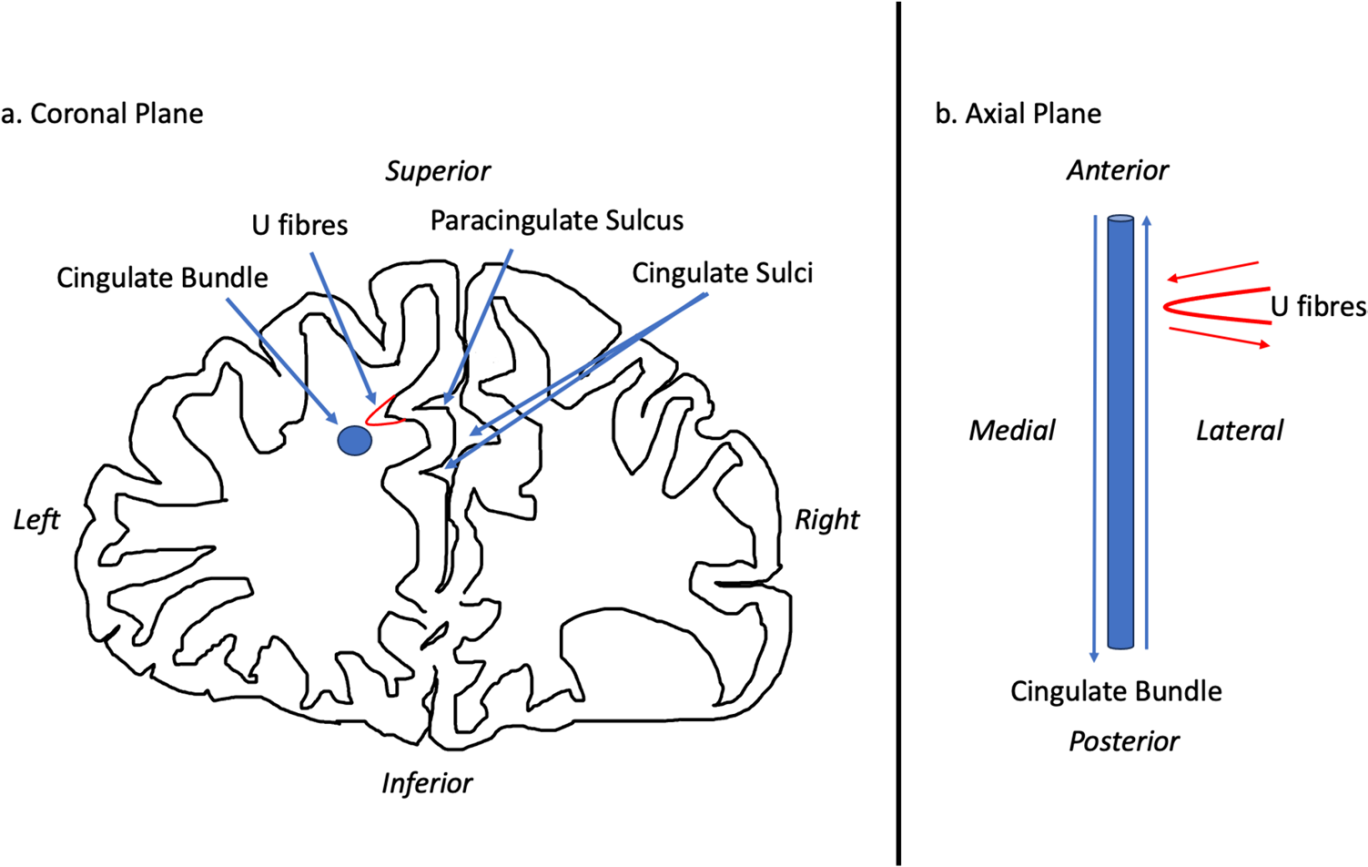
Schematic of the influence of a paracingulate sulcus on structural connectivity. **a** Schematic of a coronal cross-section of the frontal lobe of an individual with a present left and absent right Paracingulate sulcus. The blue circle represents a cross-section of the cingulum bundle along its axis. Adjacent perpendicular U-fibres are represented in red and are thought to be partially responsible for gyrification and the formation of a paracingulate sulcus. In **b** the schematic is appreciated from an axial plane. The cingulate bundle is represented in blue with blue arrows representing the predominant axial transmission along the length of the bundle. U fibres are represented in red with transmission along fibres represented by red arrows. Transmission along the cingulate bundle and adjacent U-fibres occur perpendicular to one another. Where there are increased U-fibres, such as in the presence of a paracingulate sulcus we may assume that U-fibres adjacent to the cingulate bundle may be attributed to the bundle increasing its volume and radial diffusivity increases whilst decreasing it’s fractional anisotropy

In the context of gyrification theories (Van Essen 1997, 2020) we suggest that U-fibres, (short association fibres connecting adjacent gyri displaying a complex orientation relative to major long-white mater tracts) present in greater densities in individuals with a left PCS relative to those without may have influenced tract segment metrics. This suggestion is grounded by three principles: (1.) Inclusion of U-fibres in large tracts, referred to as a transverse inaccuracy contributes to increase the tract volume within a larger white matter tract and effect diffusivity (Jbabdi and Johansen-Berg 2011). (2.) U-fibres have lower orientational coherence resulting in lower FA values. Where U-fibres are incorporated into a major tract the overall orientational coherence therefore becomes lessened resulting in a lower FA. (3.) U-fibres follow the pattern of cortical folding and as such are orientated perpendicularly to the axonal fibres of the cingulum bundle (Movahedian Attar et al. 2020) U-fibre orientation and microstructure may therefore contribute to the observed increased RD in the CBP as water molecules diffuse more freely in a radial direction with respect to the CBP proper. Figure 8 illustrates a schematic representation of these findings.

It is important to state that U-fibres are challenging to image and categorise due to their short length, size, and complex trajectories and though these metrics suggest their presence, a comprehensive assessment is indicated requiring ultra-high-resolution acquisitions as well as advanced imaging tractography methods specifically designed to identify and map U-fibres, which were not available in the present study.

In the context of the current literature our findings suggest that fibres impacted topographically by the presence of a PCS are more likely those of the cingulate bundle as suggested by Wu et al 2016 (Wu, Sun et al. 2016) than a segment of the SLF as suggested by Komaitis et al 2019 (Komaitis et al. 2019). Tract segmentation analyses are however limited in comparison to dissection techniques by spatial resolution. Furthermore, the SLF-I is large with a significant proportion of the tract located outside of the region of the expected location of the PCS. Thus the null result in in this analysis of this tract may be explained by the relatively minor contribution of PCG fibres to the SLF-I. Further study may consider restricting analysis to the anterior division of the SLF-I (as described by Komaitis et al 2019; Komaitis et al. 2019)) or to a tract ROI overlapping the expected region of the PCS. Similarly, standardised criteria for the classification of the PCS includes sulci which overlap the AC, the midcingulate (MC) or both the AC and the MC. As these regions are cytoarchitecturally and functionally different (Vogt 2019; Palomero-Gallagher et al. 2009) further study may consider sub-classifying PCS according to their presence within these regions, especially considering that our results were strongest in the CBG which primarily overlaps the AC.

Lastly, it should be acknowledged that whilst the tract segmentation analysis was exploratory, some of the obtained significant results would not have survived correction for multiple comparisons.

Contrary to our hypothesis right PCS presence was not associated with rsFC in the predefined SN or DMN as assessed according to our seed-based rsfMRI approach. In turn the Visual network which acted as an off-site control in this study identified no group difference in rsFC suggesting that findings were not secondary to a type II error. We believe these negative findings reflect the theory driven methodology in which network connectivity was analysed in accordance with parcellations corresponding to predefined networks mapped to MNI space. This method was coarse and reliant on consistent network topography between source networks used to derive the predefined networks and networks of individuals in our cohort regardless of PCS presence. Future seed-based study in this field may consider analysing network connectivity in networks derived directly form a voxel-based analysis of the same cohort.

The medial frontal lobe ROI corresponding with the location of the PCS was highly connected to regions of the cingulum, insula, frontal, temporal, and parietal cortex as well as the thalamus, caudate, pallidum, putamen, hippocampus, and amygdala, key components of the DMN and SN, in accordance with standard literature (Catani and Thiebaut de Schotten 2012). In the left hemisphere large-scale connectivity differences were observed revealing a significant network component with greater rsFC in individuals with an absent left PCS relative to those with a present left PCS. This component comprised the left and right anterior cingulate and frontal medial orbitofrontal gyrus with more extended connections to the left and right frontal superior medial gyrus and the posterior cingulum, as well as scattered connections to subcortical structures; the left amygdala, the right posterior hippocampus, and left thalamus. The increase in connectivity identifies alternate functional architecture in individuals with an absent left PCS, where distributed network nodes are enlisted creating an alternate specialisation profile with auxiliary processing power drawn from more distal regions outside of the anterior cingulate. With consideration of the findings from the tract segmentation analyses we suggest that a more dispersed network may become operational where a highly localised network (presumed to exist where a PCS is present) is not present. In turn, cognitive advantages reported in the literature (Fornito et al. 2004; Whittle et al. 2009a, b; Borst et al. 2014a, b, Cachia et al. 2017) associated with the presence of a left PCS may be underpinned by an efficient highly localised network dependent on U-fibres rather than a well organised cingulum bundle. These observations are in line with and provide further evidence for the tension-based morphogenesis theory of cortical folding (Van Essen 1997) and support the notion that well interconnected brain regions display strong patterns of functional connectivity (Segall et al. 2012; van den Heuvel et al. 2009). Extending this concept to disease, we speculate that a highly connected localised network existing in the presence of a PCS may explain why absence of a left PCS, a neurodevelopmental variation, has been associated with both schizophrenia and OCD (Yücel et al. 2002; Shim et al. 2009). Studies exploring these hypotheses in these disease groups are indicated in order to provide evidence for this theory. Furthermore, it is known that AC gyral variability affects gyral volume and thus should be taken into account in the study of relevant diseases (Fornito et al. 2006). Here we demonstrate that this is also the case for structural anatomy.

The impact of sulcal morphology on rsFC has been explored previously. In the neighbouring ventromedial prefrontal cortex, similar to in the present study, Lopez-Persem et al 2019 report an impact of sulcal variability on rsFC topography (Lopez-Persem et al. 2019). Furthermore, this study identified a correlation between ventromedial prefrontal cortex resting state activity and sulcal depth, with greater activity observed in the proximity of sulci, which is in keeping with the tension-based morphology theory of sulcation and potentially represents a highly connected local network dependent on high U-fibre density.

Contrary to findings of the present study, Fedeli et al 2020 explored rsFC with respect to PCS presence using a seed-based approach and did not identify an association between rsFC and left PCS presence (Fedeli et al. 2020). Similarly to this study however, an association between individuals with absent PCS and enhanced long-distance rsFC was identified in Fedeli et al 2020. Albeit, this connection was formed with the cerebellum, a region not identified as a highly connected region to the medial frontal lobe ROI in our study and therefore not investigated further for connectivity differences according to PCS presence in the second part of our voxel-based analysis. Fedeli et al 2020 also report additional associations between whole brain PCS patterns (bilateral PCS status) and distinct profiles of rsFC. These findings include decreased connectivity in the insula in those with bilaterally absent PCS compared to those with bilaterally present PCS, extensive decreased patterns of long-distance rsFC to the bilateral occipital cortices, right temporo-occipital and cerebellar regions in individuals with a bilaterally absent PCS compared to those with a rightward dominant pattern and increased connectivity with the angular gyrus, insular and central opercular cortex in individuals with bilaterally present PCS compared to those with leftward dominant patterns (Fedeli et al. 2020). Findings from this study indicate a functional effect of gyral variation but lack a proposed unifying mechanistic theory.

Conversely, employing a different methodology, Loh et al. 2018 observed no association between paracingulate sulcal presence and rsFC in left hemisphere motor ROIs within the midcingulate (Loh et al. 2017). RsFC analysis in relation to whole brain PCS pattern was not performed in the present study due to powering though further study in this field should investigate this topic in order to identify if findings from Fedeli et al 2020 may be replicated.

RsfMRI data in the present study was obtained at a spatial resolution of 6 × 6x6 mm voxel connectivity with 5000 voxels. Though collection of data at this resolution allows for timely attainment of data from large cohorts a potential limitation is that this degree of spatial resolution does not provide the sensitivity required to identify functional connectivity differences generated by highly localised networks. Furthermore, it is quite probable that both individuals with a present and absent PCS have inherently high intra-connectivity in a highly localized functional map within the targeted ROI which is not reflected in groupwise testing. Where feasible alternative fMRI techniques may be utilised for further exploration of this theory in future study.

### Laterality

We did not observe significant structural or functional connectivity differences in the right hemisphere consistent with those found in the left hemisphere. This was unexpected and in relation to the tension-based morphogenesis theory, presence of a PCS should be associated with local increased U-fibre density bilaterally. That considered, an asymmetry of the cingulum has been identified in diffusion based studies such that volume (Takao et al. 2013) and FA, along the length the anterior cingulum displays a marked left‐greater‐than‐right asymmetry (Gong et al. 2005; Park et al. 2004), notably however, these studies did not account for PCS presence. Furthermore, superficial white matter, U-fibres (which contribute 90% of the total white matter fibres to the human brain) are known to display an asymmetrical distribution, with diffusivity metrics indicative of increased left hemisphere U-fibre structural integrity in the frontal, temporal, and parietal regions of healthy individuals (Movahedian Attar et al. 2020). With consideration of these data, the lateralisation identified in this study may be represented by an increased left-to-right hemisphere asymmetry in U-fibre density.

Lateralising findings in analyses of connectivity in the predefined functional networks were not identified here, however the SN is known to be organizationally dominant in the right hemisphere (Seeley et al. 2007; Zhang et al. 2019) with multimodal structural and functional imaging studies(Cauda et al. 2011; Zhang et al. 2019; Seeley et al. 2007; Zhang et al. 2019) identifying stronger and broader intrinsic functional network couplings in the right compared to left dorsal ACC. In turn, the right hemisphere SN has been identified to exhibit much weaker disassortativity (the degree of connection between nodes with low numbers of connections and nodes with high numbers of connections) than that of the left hemisphere (Lim et al. 2019). Similarly, these observations may also be attributed to U-fibres density asymmetries between hemispheres.

## Summary

These results identify a novel association between sulcation, a neurodevelopmentally derived gross anatomical feature and altered structural and functional connectivity in a healthy adult population. Furthermore, they provide evidence of a link between structural and functional connectivity and a plausible explanation of how cognitive advantages of a paracingulate sulcus may be mediated by a highly connected local functional network reliant on short association fibres. The findings also have importance for understanding the neuropsychological aspects of this anatomical variation, and for understanding pathophysiology of the diseases in which this variation has a role. Additional work in this field utilizing multimodal imaging techniques in adequately sized cohorts is indicated to confirm results presented here, provide evidence to support our rational and investigate structural and functional connectivity with respect to PCS patterns.

## Supplementary Information

Below is the link to the electronic supplementary material.

Supplementary file1 (DOCX 11376 KB)

## Data Availability

No datasets were generated or analysed during the current study.
