# Supplementary material for: Structural and functional connectivity associations with anterior cingulate sulcal variability
Source: Brain Struct Funct. 2024 Jun 20;229(7):1561–76. doi: 10.1007/s00429-024-02812-5 (PMC11374863; doi:10.1007/s00429-024-02812-5)
Supplement: Supplementary file 1 — Supplementary file1 (DOCX 11376 KB) [file 429_2024_2812_MOESM1_ESM.docx]

**Anterior Cingulate Sulcal variability and Salience Network Connectivity: Supplementary Material**

**Running Title: Anterior Cingulate Sulcal variability and Salience Network Connectivity**

**Authors:**

Luke Harper, MD^1^; Olof Strandberg, PhD^1^; Nicola Spotorno, PhD^1^; Markus Nilsson, PhD^2^; Olof Lindberg, PhD^3^; Oskar Hansson, MD, PhD^1,4*^; Alexander F Santillo, MD, PhD^1*^

^1^Clinical Memory Research Unit, Department of Clinical Sciences, Lund University, Malmö, Sweden

^2^Diagnostic Radiology, Faculty of Medicine, Department of Clinical Sciences, Lund, Sweden

^3^Division of Clinical Geriatrics, Karolinska Institute, Stockholm, Sweden

^4^Memory Clinic, Skåne University Hospital, Lund, Sweden

*Equal contribution

**Corresponding Author**

Luke Harper^1^

Medical Sciences, Neuroscience, Lund University, Sölvegatan 19, Lund, Sweden, 22100

[l.harper@doctors.org.uk](mailto:l.harper@doctors.org.uk)

0046704449031

**Materials and Methods**

**CSF amyloid-ß42/40 ratio**

As described in Janelidze et al 2016^1^ CSF Aβ42 and Aβ40 were measured using the Elecsys® CSF electrochemiluminescence immunoassay (Roche Diagnostics) and Roche NeuroToolKit, respectively in all but one participant who’s CSF Aβ42 and Aβ40 were quantified using MSD assays. Aβ status (negative/positive) was determined using CSF Aβ42/Aβ40 ratio based a threshold of 0.08 (Roche). For MSD CSF Aβ42/Aβ40, the threshold of <0.077 was defined using mixture modelling.

**White matter hyperintensities**

Total white matter hyperintensities (WHMs) volumes were identified using an automated method WMH segmentation with Sequence Adaptive Multimodal SEGmentation (SAMSEG), which is an unsupervised, fully-automated, contrast-based method of segmentation freely available through FreeSurfer (version 7.2)^2^ ^3^. SAMSEG performs lesion segmentation in the context of whole brain modelling, incorporating both T1- and T2-weighted images as inputs. SAMSEG employs unsupervised Gaussian mixture modelling to automatically group together voxels with similar intensities and perform voxel segmentation^4^.

**Paracingulate Sulcus Measurement and Classification Criteria**

Images were imported into MANGO (Multi-image Analysis GUI, v 4.0, <http://ric.uthscsa.edu/mango/mango.html>, The University of Texas Health Science Centre) software and prepared, aligning the x-axis in the sagittal plane with the bicommissural line (AC–PC). Further y- and z-axes rotational corrections were performed in order to ensure optimal orientation for analysis PCS presence. Manual PCS classification was performed radiographically according to a protocol adapted from Garrison’s established protocol for PCS classification ^5^, used and described in Harper et al 2022^6^. A binary sulcation classification was utilised where the PCS was categorized as either “present” (≥20 mm) or “absent” (≤19 mm) as is standard amongst PCS classification protocols.^5, 7-10^. The cingulate sulcus (CS) is identified 4 mm laterally from the midline (x = 0). The PCS is identified as the sulcus running predominantly horizontally, dorsal and parallel to the CS. The PCS is measured from its anterior limit, identified as the point at which the sulcus begins to move posteriorly and parallel to the CS from an imaginary line perpendicular to the AC–PC line ^11^. The PCS is measured from this point using MANGO’s “Trace Line” function until its end point, the point where the sulcus is interrupted by a distinct predominantly vertical gyri deemed non-PC in nature. The PCS may fall outside of the first quadrant but must originate in the first quadrant on a sagittal plane where x0, y0 marks the point of the anterior commissure. Garrisons protocol^5^ was modified in the present study and Harper et al 2022^6^, such that discontinuous PCS lacking an individual segment ≥20 mm in length were classified as “absent”. This decision was made as raters in this study found measurement of these structures to be inconsistent and unreliable in pre-study rater training. In addition, present PCS were required to be visible on four consecutive sagittal slices (a total depth of >4mm). Measurement was performed on the sagittal slice where the anteroposterior PCS length was shortest of the four longest consecutive sagittal slices where the PCS could be identified. This adaption meant that sulcation length was maintained throughout a depth of four sagittal slices which we believe to have increased the reliability of detection of a true PCS. Additionally, present PCS were subclassified as “prominent” where their length exceeded 40mm, as performed by others^5^ ^11^. Sulcation ratings were performed by two raters, LH and AS, who were blinded to individuals’ clinical and demographic data. Disagreement between raters was resolved by consensus. An intra-rater agreement of 98.33%, *Cohens Kappa* 0.96 and an inter-rater agreement of 94.44%, *Cohens Kappa* 0.85 has been reported in reliability studies conducted by these raters using this protocol ^12^.

**Tractography**

Following pre-processing of diffusion MRI scans white matter tracts were delineated using two automated methods, TractSeg^13^ and Xtract^14^. Data was quality controlled as such individuals (n=1) with an average total motion [mm] value +/- 3 standard deviations from the mean were removed prior to analysis. White matter tract integrity was controlled, and data was examined for large outliers (values < quartile 1 + 3x IQR or > quartile 3 + 3x IQR) according to total white matter volume [ml] and the percentage of voxels in the total brain mask where mean diffusivity (MD) was > 0.003 10-6m2s-1, an indicator of relative ventricle size. No such individuals were identified. As a further quality control measure, whole brain total white matter volume, ventricular size and average motion was analysed for group differences depending on hemispheric PCS status, identifying similarity between groups.

**Seed-based analysis Networks of Interest**

Networks were defined geographically according to network parcel locations defined by the Schaefer 200 parcel 7 network atlas^15^, these included; The Salience/Ventral Attention Network (Left hemisphere: parcels 44 – 54, Right hemisphere parcels: 148 to 158), the DMN (Left hemisphere parcels: 74 – 100, Right hemisphere parcels 182 to 200) and the Visual network (Left hemisphere: parcels 1 – 14, Right hemisphere: parcels 101 to 115).

**Supplementary Fig. 1. Voxel-based analysis medial frontal lobe regions of interest**


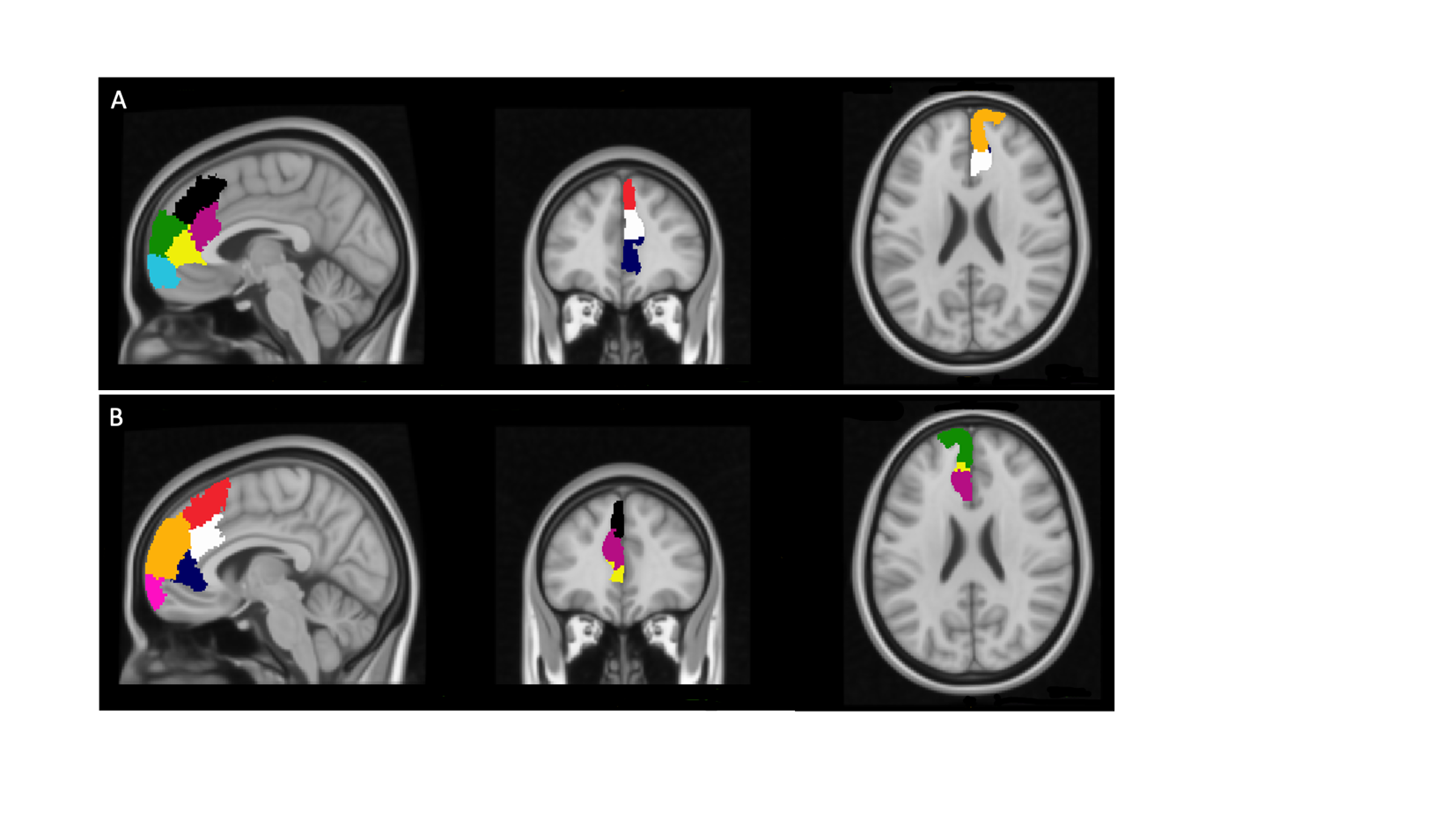


Right and left hemisphere medial frontal lobe regions of interest (ROI) overlapping the paracingulate sulcus utilised in the voxel-based functional connectivity analysis, projected in MNI 152 space^16^ at 1mm spatial resolution. Panel A displays the right hemisphere ROI projected, from left to right on sagittal, coronal, and axial slices. The right hemisphere ROI consists of parcels 161(Pink), 180 (White), 181 (Red), 192 (Blue) and 194 (Orange) of the Schaefer 200 parcels 7 network atlas^17^. Panel B similarly displays the left hemisphere ROI which consists of parcels 86 (Light Blue), 88 (Yellow), 89 (Dark green), 90 (Purple) and 92 (Black).

**Supplementary Figure 2. Temporal cingulum bundle tract mean and radial diffusivity by left paracingulate sulcal presence**


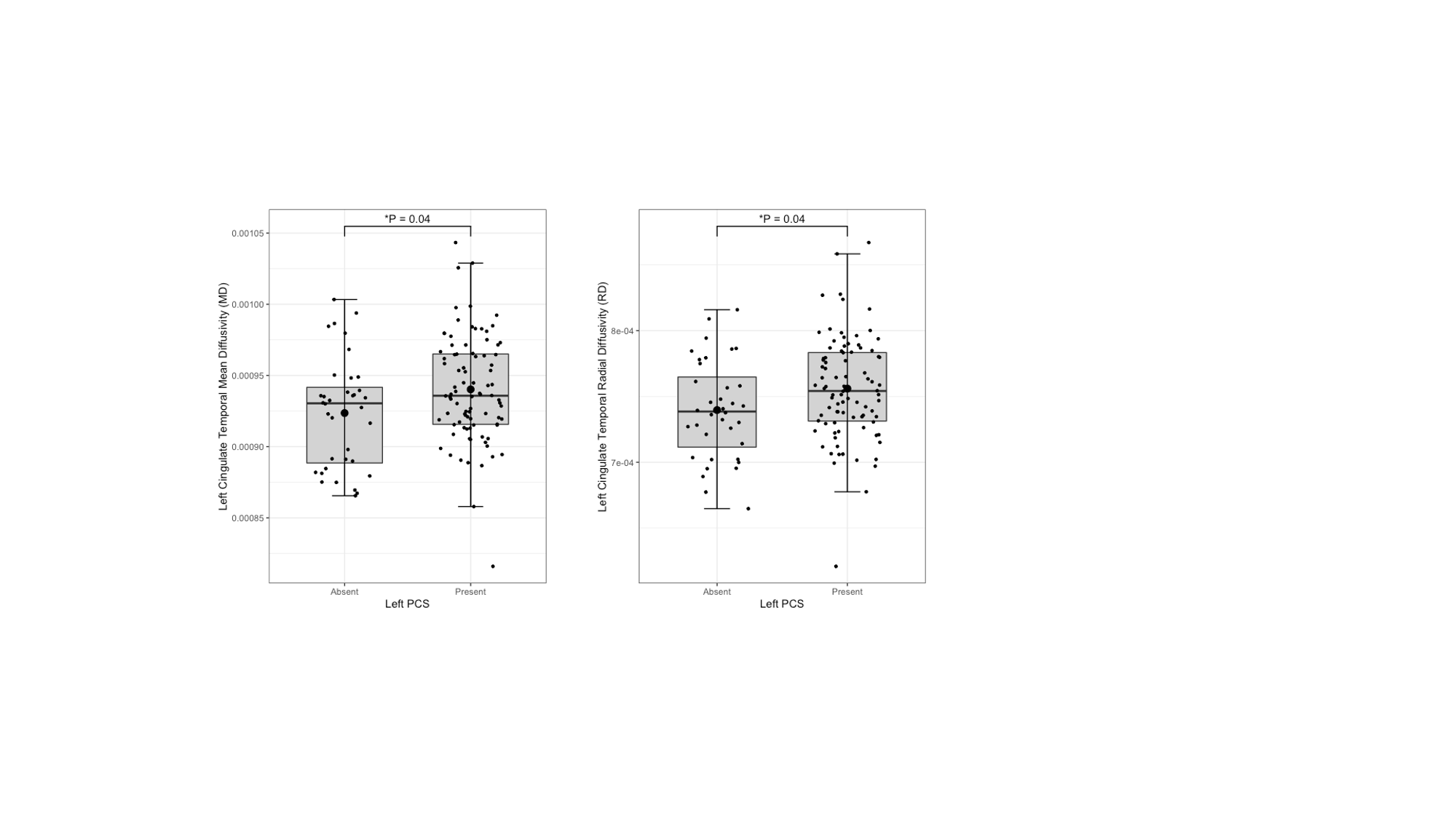


**Sup. Fig. 2.** Box plot displaying left temporal cingulum bundle (CBT) [Xtract] mean diffusivity (left panel) and radial diffusivity (right panel) by left Paracingulate sulcal presence. Black dots represent individuals, n = 125. Thick horizontal black lines represent group median values. Larger black dots represent group mean values. Boxes extend from the 25^th^ to the 75^th^ percentile, horizontal black lines within the boxes denote median values. P-values (P) of general linear models corrected for age, sex and handedness are displayed over the box plots. * Significance at P= < 0.05. ** P= < 0.01.

**Supplementary Figure 3. Summed connectivity of the significantly differing network component by left paracingulate sulcal presence**

*
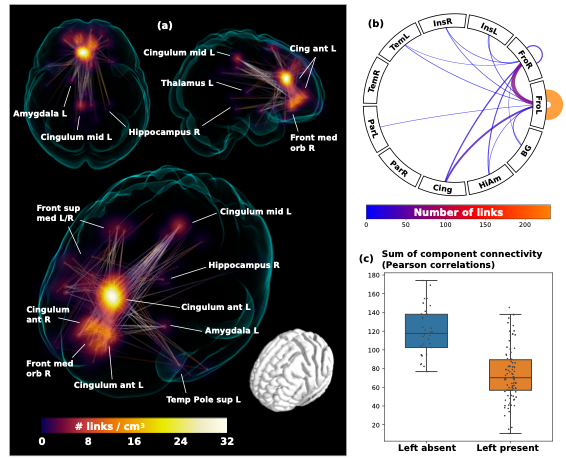
*

**Sup.Fig.3.** Box plot displaying the summed connectivity of the significantly differing network component for individuals with a absent left paracingulate sulcus (Left absent, blue) relative to individuals with a present left paracingulate sulcus (Left present, orange), n = 127. Black dots represent individuals. Boxes extend from the 25^th^ to the 75^th^ percentile, horizontal black lines within the boxes denote median values. P = 0.01 controlled for family-wise error rate.


**Supplementary Tables**

**Supplementary Table 1. Seed-based Functional Connectivity Results**

| **Network** | **Dependent Variable** | **PCS Contrast** | **Confidence Interval** |
| --- | --- | --- | --- |
| Salience Network, left | Left Paracingulate Presence | β = -0.02, *P* = 0.07 | -0.05 - 0.002 |
| Salience Network, right | Right Paracingulate Presence | β = -0.01, *P* = 0.48 | -0.03 - 0.02 |
| Salience Network, right & left | Left Paracingulate Presence | β = -0.03, *P* = 0.06 | -0.05 - 0.0009 |
| Salience Network, right & left | Right Paracingulate Presence | β = -0.02, *P* = 0.14 | -0.04 - 0.006 |
| Default Mode Network, left | Left Paracingulate Presence | β = -0.01, *P* = 0.54 | -0.03 - 0.02 |
| Default Mode Network, right | Right Paracingulate Presence | β = - -0.0001, *P* = 0.99 | -0.02 - 0.02 |
| Default Mode Network, right & left | Left Paracingulate Presence | β = -0.01, *P* = 0.45 | -0.03 - 0.02 |
| Default Mode Network, right & left | Right Paracingulate Presence | β = -0.001, *P* = 0.93 | -0.02 - 0.02 |
| Visual Network, left | Left Paracingulate Presence | β = -0.02, *P* = 0.22 | -0.06 - 0.01 |
| Visual Network, right | Right Paracingulate Presence | β = 0.002, *P* = 0.91 | -0.03 - 0.03 |
| Visual Network, right & left | Left Paracingulate Presence | β = -0.02, *P* = 0.22 | -0.06 - 0.01 |
| Visual Network, right & left | Right Paracingulate Presence | β = 0.003, *P* = 0.86 | -0.03 - 0.04 |

General Linear Models for differences in predefined network functional connectivity according to ipsilateral paracingulate sulcal presence. All models are corrected for age, sex, and handedness.

**References**

1. Janelidze S, Zetterberg H, Mattsson N, et al. CSF Aβ42/Aβ40 and Aβ42/Aβ38 ratios: better diagnostic markers of Alzheimer disease. *Ann Clin Transl Neurol*. Mar 2016;3(3):154-65. doi:10.1002/acn3.274

2. Puonti O, Iglesias JE, Van Leemput K. Fast and sequence-adaptive whole-brain segmentation using parametric Bayesian modeling. *NeuroImage*. 2016/12/01/ 2016;143:235-249. doi:<https://doi.org/10.1016/j.neuroimage.2016.09.011>

3. Cerri S, Puonti O, Meier DS, et al. A contrast-adaptive method for simultaneous whole-brain and lesion segmentation in multiple sclerosis. *NeuroImage*. 2021/01/15/ 2021;225:117471. doi:<https://doi.org/10.1016/j.neuroimage.2020.117471>

4. Ferris JK, Lo BP, Khlif MS, Brodtmann A, Boyd LA, Liew SL. Optimizing automated white matter hyperintensity segmentation in individuals with stroke. *Front Neuroimaging*. 2023;2:1099301. doi:10.3389/fnimg.2023.1099301

5. Garrison JR, Fernyhough C, McCarthy-Jones S, Haggard M, Simons JS. Paracingulate sulcus morphology is associated with hallucinations in the human brain. *Nat Commun*. Nov 17 2015;6:8956. doi:10.1038/ncomms9956

6. Harper L, Lindberg O, Bocchetta M, et al. Prenatal Gyrification Pattern Affects Age at Onset in Frontotemporal Dementia. *Cerebral Cortex*. 2022;doi:10.1093/cercor/bhab457

7. Ono M, Kubik S, Abernathey CD. Atlas of the cerebral sulci. 1990;

8. Del Maschio N, Sulpizio S, Fedeli D, et al. ACC Sulcal Patterns and Their Modulation on Cognitive Control Efficiency Across Lifespan: A Neuroanatomical Study on Bilinguals and Monolinguals. *Cereb Cortex*. Jul 5 2019;29(7):3091-3101. doi:10.1093/cercor/bhy175

9. Yücel M, Stuart GW, Maruff P, et al. Paracingulate morphologic differences in males with established schizophrenia: a magnetic resonance imaging morphometric study. *Biol Psychiatry*. Jul 1 2002;52(1):15-23. doi:10.1016/s0006-3223(02)01312-4

10. Le Provost JB, Bartres-Faz D, Paillere-Martinot ML, et al. Paracingulate sulcus morphology in men with early-onset schizophrenia. *Br J Psychiatry*. Mar 2003;182:228-32. doi:10.1192/bjp.182.3.228

11. Yücel M, Stuart GW, Maruff P, et al. Hemispheric and gender-related differences in the gross morphology of the anterior cingulate/paracingulate cortex in normal volunteers: an MRI morphometric study. *Cereb Cortex*. Jan 2001;11(1):17-25. doi:10.1093/cercor/11.1.17

12. Harper L, Lindberg O, Bocchetta M, et al. Prenatal gyrification pattern affects age at onset in frontotemporal dementia. *Cerebral Cortex*. 2022;32(18):3937-3944. doi:10.1093/cercor/bhab457

13. Wasserthal J, Neher P, Maier-Hein KH. TractSeg - Fast and accurate white matter tract segmentation. *Neuroimage*. Dec 2018;183:239-253. doi:10.1016/j.neuroimage.2018.07.070

14. Warrington S, Bryant KL, Khrapitchev AA, et al. XTRACT - Standardised protocols for automated tractography in the human and macaque brain. *Neuroimage*. Aug 15 2020;217:116923. doi:10.1016/j.neuroimage.2020.116923

15. Schaefer A, Kong R, Gordon EM, et al. Local-Global Parcellation of the Human Cerebral Cortex from Intrinsic Functional Connectivity MRI. *Cerebral Cortex*. 2017;28(9):3095-3114. doi:10.1093/cercor/bhx179

16. Grabner G, Janke AL, Budge MM, Smith D, Pruessner J, Collins DL. Symmetric atlasing and model based segmentation: an application to the hippocampus in older adults. Springer; 2006:58-66.

17. Schaefer A, Kong R, Gordon EM, et al. Local-Global Parcellation of the Human Cerebral Cortex from Intrinsic Functional Connectivity MRI. *Cereb Cortex*. Sep 1 2018;28(9):3095-3114. doi:10.1093/cercor/bhx179
